# A Review of the Use of Coconut Fiber in Cement Composites

**DOI:** 10.3390/polym15051309

**Published:** 2023-03-06

**Authors:** Flávia Regina Bianchi Martinelli, Francisco Roger Carneiro Ribeiro, Markssuel Teixeira Marvila, Sergio Neves Monteiro, Fabio da Costa Garcia Filho, Afonso Rangel Garcez de Azevedo

**Affiliations:** 1 LAMAV-Advanced Materials Laboratory, Campos dos Goytacazes, State University of the Northern Rio de Janeiro, Campos dos Goytacazes 28013-602, RJ, Brazil; 2 Civil Engineering Graduate Program, Federal University of Rio Grande do Sul (UFRGS), Porto Alegre 90035-190, RS, Brazil; 3 CRP-Rio Paranaíba Campus, UFV-Federal University of Viçosa, Rio Paranaíba 38810-000, MG, Brazil; 4 Department of Materials Science, IME—Military Institute of Engineering, Rio de Janeiro 22290-270, RJ, Brazil; 5 LECIV-Civil Engineering Laboratory, UENF-State University of the Northern Rio de Janeiro, Campos dos Goytacazes 28013-602, RJ, Brazil

**Keywords:** vegetable fibers, coir fiber, cementitious matrix composite

## Abstract

The use of plant fibers in cementitious composites has been gaining prominence with the need for more sustainable construction materials. It occurs due to the advantages natural fibers provide to these composites, such as the reduction of density, fragmentation, and propagation of cracks in concrete. The consumption of coconut, a fruit grown in tropical countries, generates shells that are improperly disposed of in the environment. The objective of this paper is to provide a comprehensive review of the use of coconut fibers and coconut fiber textile mesh in cement-based materials. For this purpose, discussions were conducted on plant fibers, the production and characteristics of coconut fibers, cementitious composites reinforced with coconut fibers, cementitious composites reinforced with textile mesh as an innovative material to absorb coconut fibers, and treatments of coconut fiber for improved product performance and durability. Finally, future perspectives on this field of study have also been highlighted. Thus, this paper aims to understand the behavior of cementitious matrices reinforced with plant fibers and demonstrate that coconut fiber has a high capacity to be used in cementitious composites instead of synthetic fibers.

## 1. Introduction

The scarcity of non-renewable raw materials and the incorrect disposal of solid waste in the environment drive society toward more sustainable buildings that respect nature and the atmosphere. Construction materials deteriorate over time due to innumerable problems, be they natural causes, design, component quality, and/or execution errors, which can lead to a partial or total collapse of the construction system. Several types of concrete are developed from the better design of materials and mixtures for specific applications, but they have low tensile strength and ductility. 

According to Afroughsabet et al. [1], these problems are caused by micro-cracks that develop by shrinkage due to the high binder content and a low water/cement ratio in high-strength concretes, for example. These microcracks cause the non-linear behavior of concrete at low stress levels and its volumetric expansion before failure. However, to obtain better knowledge about cracks in concrete structures, not only at an early age, it is necessary to understand the types of cracks/fractures, trends of occurrence, causes, and preventive measures to combat them [2]. New strengthening methods have been the target of investigations due to this disadvantage in concrete and the search for environmentally friendly materials for civil construction.

One of the potential alternatives is the incorporation of natural fibers into the cement matrix, replacing synthetic fibers and steel. These fibers can reduce production costs by increasing the tenacity, lightness, mechanical strength, impact resistance, and biodegradability of cement-based composites. The matrix distributes part of the load to the fibers during loading before any cracks appear [3]. The fibers confer better mechanical properties to the concrete due to their higher tensile strength and elasticity modulus than the cement matrix. If this does not occur, there is a failure of adhesion of the fibers to the composite [4,5].

In addition to the contributions mentioned above, plant fibers can be recycled, are renewable, are locally available, do not require energy for their production, and do not increase the carbon footprint of concrete compared to synthetic fibers. Some studies have reported that adding 1% steel fiber can increase the cost of concrete by more than 90% and the carbon footprint by another 50% [6,7]. 

As disadvantages of natural fibers, they have higher variability in physical and mechanical properties, lower durability, low resistance to microbial attack, a tendency to form aggregates during processing that may cause poor adhesion of the fiber to the matrix, and low moisture resistance. The variety of properties depends mainly on the plant species, growing conditions, fiber extraction method, fiber cell geometry, each type of cellulose, and its degree of polymerization. It is important to note that the hemicelluloses and the lignin give the fiber its stiffness [8,9]. However, these problems can be overcome by modifying the fiber surface with chemical, physical, and mechanical treatment methods [10,11,12,13]. These properties are controlled by the wettability nature of the fiber, regulating adhesion behavior and mechanical strength gain [13].

The properties of cement-based composites depend on several factors, such as the type and amount of fiber [14,15], the fiber length for better adhesion to the matrix [16], fiber treatment [4,17], and the correct dispersion of the fiber in the matrix [9]. As for the mechanical requirements of composites, tensile, flexural, and impact strengths are the most analyzed tests in several studies [5,16,18,19,20,21].

Given this context, this review aims to establish a parallel between the physical and mechanical performance of cementitious composites reinforced with coconut fibers, highlighting the main parameters that improve the quality of materials produced with these fibers. This research differs from others due to its focus on effectively investigating the use of coconut fibers in cementitious composites, clarifying that these coconut fibers can become textile meshes as another reinforcement design, and highlighting the best treatments of coconut fibers that do not cause damage to the properties of cement-based composites.

## 2. Vegetable and Coconut Fibers

According to Dhandhania and Sawant [22], using fibers as reinforcement started in ancient Egyptian times with flax fiber. Later came asbestos, but this material presents health risks, making its use unfeasible. Then, in the late 1960s, steel, glass, and carbon fibers started to be used, and several studies were dedicated to analyzing the use of natural fibers to replace synthetic fibers.

Plant fibers are the most commonly used fibers in the industry for different applications and to replace synthetic materials and non-renewable resources. Among their advantages over synthetic fibers are accessibility, low cost, low density, a good modulus-to-weight ratio, high acoustic insulation, lower industrial energy consumption, decomposition, and carbon “free” (that is, when they are composted or incinerated, they release the same amount of carbon dioxide consumed during their development). On the other hand, natural fibers have some limitations due to their less reliable properties in nature and their excellence.

Plant fibers, composed mainly of cellulose, are also called cellulosic or lignocellulosic fibers and are made up of individual cells composed of microfibrils arranged in layers with different thicknesses and angles of orientation. These microfibrils are rich in cellulose, a long-chain plant polymer, and are surrounded by a "matrix" of hemicellulose and lignin, amorphous fiber components. Pectins permeate the cellulose-hemicellulose network. Another important constituent of the walls is lignin, a hydrophobic substance impregnated mainly in the layers near the surface and which has a supporting function [23].

Cellulose, considered to be the main constituent of living organisms, is a linear polymer formed by cellobiose units (glycosyl-glucose disaccharide). Its chains come together in the constitution of the plant structure forming crystalline regions, due to the formation of intra and intermolecular hydrogen bridges (generating rigidity and organized three-dimensional arrangement to the chains), interspersed by amorphous regions. The alignment of the molecules leads to the formation of micelles (crystals of cellulose molecules on the order of angstroms), which unite to form the microfibrils. The microfibrils unite to form the macrofibrils, which together with the lignin form the cell wall [24].

Cellulose fibers in a matrix of hemicellulose, lignin, and proteins make up the different layers of natural fiber. Cellulose is the main structural component that provides a natural fiber with strength and stability and is resistant to strong alkalis but is easily hydrolyzed by acid to form water-soluble sugars. Hemicellulose is a branched polymer made of several polysaccharides. Compared to cellulose, hemicellulose contributes little to fiber stiffness and strength, is very hydrophilic and soluble in alkalis, and is readily hydrolyzed in acids. As for lignin, it is an amorphous, heterogeneous mixture of aromatic polymers and phenyl propane monomers and is responsible for natural fibers’ compressive strength and compressive stiffness [25].

The fiber cells are 10 to 25 μm in diameter and contain four layers of microfibrils. The outermost layer, the primary layer, and the microfibrils have a cross-linked structure. Secondary layers can be found internally. The secondary layer S1 also has a cross-linked structure. In the secondary layer S2, the microfibrils are aligned at an angle θ, with respect to the longitudinal axis of the cell, in a spiral. This angle can vary in the different layers. The innermost secondary layer, S3, exhibits a similar configuration. Inside the cell is a central cavity called the lumen, with a dimension of 5–10 to 10 μm [24,25,26].

According to Lau et al. [27], natural fibers have complicated structures from a microscopic perspective. The lumen core is surrounded by different cell wall layers with different microfibril orientations, which give strength to the fiber subjected to different loads. The microfibril angle governs the increase in tensile strength of natural fibers, and the elongation at break increases with the microfibril angle [27]. The authors also mention that microfibrils are not identical, as they are composed of crystalline and amorphous regions. The former determines the fiber strength, while the latter is relatively soft and formed by irregular molecular chains. Table 1 compares the properties of some different natural fibers with different microfibril orientations.

According to Lertwattanaruk and Suntijitto [40], the chemical compositions of natural fibers are different due to cultivation methods and environmental conditions such as soil, water, air, and chemicals used. Table 2 presents the chemical components of the industry’s most commonly used dried plant fibers with the average amount (% by weight) of the compositions.

Cellulosic fibers have a hydrophilic nature (affinity for water) under natural conditions [24]. The moisture content in the fibers can negatively influence the mechanical behavior of natural fiber composites. One way to improve this mechanical behavior is through surface modification of the fiber by physical or chemical methods. Table 3 illustrates the equilibrium moisture content of natural fibers considering a relative humidity of 65% and a temperature of 21 °C, according to Rowell [41].

The world production of coconut was estimated at approximately 60.5 million tons in 2021, with the leading producers being Indonesia, responsible for 28% of production, followed by the Philippines with 24% and India with 22%. Brazil, in turn, ranks fourth, producing 4.2% [42]. Under a Brazilian scenario, the country produced 1.6 million fruits in 2021, with the Northeast region accounting for 74% of this production [43]. The generation of coconut shells, a waste generated by coconut consumption, was estimated to be 70% of the solid waste collected on Brazilian beaches [44].

According to Marafon et al. [45], the coconut has an average weight of 0.9 to 1.5 kg, varying according to the coconut tree species. It is classified as a fleshy fruit with only one seed. The fruit of the coconut palm consists of liquid albumen (coconut water), solid albumen (pulp), endocarp, and the shell. The coconut shell is made up of the fibers (70%), which constitute the mesocarp of the fruit, the dust (30%), which is the filling material of the interfibrillar spaces, and the epicarp (outer shell).

Coconut fiber has a cylindrical structure (~10 to 460 µm in diameter), with a hollow area surrounded by 200 to 300 elementary fibers. An elementary fiber is a hollow cylindrical structure formed by individual fiber cells with a diameter of 10 to 20 µm and a length of approximately 1 mm [46,47,48,49]. 

Based on the maturity of the coconut, two types of fibers still exist, i.e., white or light brown fiber and dark brown fiber, which are extracted from the immature and mature coconut, respectively. The white fiber is smooth and thin [50], and the brown fiber is stronger, thicker, and has higher abrasion resistance in spite of its long growth time, making it more suitable for reinforcement [51]. 

In addition, this fiber has cellulose, hemicellulose, lignin, pectin, and minerals. Of these constituents, cellulose (22% to 44% content) is primarily responsible for fiber stability and strength, as well as lignin (40% to 45% content), which is responsible for water and fungal resistance and high flexibility [8,51]. The high flexibility of these fibers provides fracture resistance to elements reinforced with coconut fibers, allowing for no debonding of the concrete when cracking and increasing the durability of these elements.

As for the microstructure of the fiber, some studies have observed that coconut fibers “in natura” have rough surfaces [21,52,53]. Regarding thermal stability, decomposition is associated with the contents of cellulose, hemicellulose, and lignin in the fiber. Cellulose degrades between 240 and 350 °C, hemicellulose between 200 and 260 °C, and lignin, in turn, between 280 and 500 °C [54].

According to Zamora-Castro et al. [55], coconut fiber can reach 4 to 6 times more deformation than other natural fibers. In addition, coconut fiber has the highest strength among all-natural fibers. These mechanical characteristics reduce cracking, leading to coconut fiber-reinforced concrete with better flexural behavior and higher impact resistance than traditional concrete.

## 3. Coconut Fiber Reinforced Cementitious Composites

Nehvi and Kumar [56] found that the slump test revealed that concrete without fibers recorded the maximum slump value (83 mm), while concrete with 1% coconut fiber recorded the highest slump value (50 mm). The proportional increase in fiber concentration showed a decline in slump value from 50 mm to 19 mm with coconut fiber. Ahmad et al. [57] observed that the slump is affected by both dosage and length of coconut fiber, especially at lengths greater than 75 mm.

According to Srinidhi et al. [58], adding coconut fiber to concrete increased its compressive strength by 20–25% compared to the reference concrete using 0.5–1.5% fiber addition. Krishna et al. [59] and Abhishek et al. [60] also found noticeable benefits in these specified ranges. However, in lightweight concretes, Mydin et al. [61] and Zamzani et al. [62] increased the compressive strength by 15–40%.

Hwang et al. [63] developed experimental tests on coconut fiber-reinforced concrete and observed that adding 4% coconut fiber to concrete can decrease its compressive strength by about 50%. In addition, the coconut fiber-reinforced concrete’s water absorption and flexural strength can be increased by up to 33% and 50%, respectively. Based on the stress-strain curves of coconut fiber concrete, they reported that its modulus of elasticity decreased. At the same time, its maximum strain reached 6 × 10^−3^, which is two times higher than conventional concrete. Thus, coconut fiber can be used as a polymeric fiber in concrete for slabs, plates, and pavement.

Zamora-Castro et al. [55] reported that ropes composed of coconut fibers had been employed as vertical reinforcement of concrete walls to increase the stability of structures against earthquakes. They developed experimental tests with coir ropes to evaluate the tensile strength of coir ropes [64]. They proposed that the length of coir cables embedded in concrete should be greater than 200 mm. Furthermore, they observed that coconut fiber-reinforced concrete and the treatment in which coconut ropes are boiled increase the bond strength and pull-out energy. Regarding the performance of coconut fiber-reinforced concrete columns with rope tested on oscillating tables, he found that invisible degradation occurred prior to cracking these columns. In addition, a rope configuration can keep the column attached to its support after being tested. On the other hand, Wang and Chouw [65] used coconut fibers to improve concrete properties under load impact. The authors determined that coconut fiber reduced fragmentation and cracking of coconut fiber-reinforced concrete.

Rumbayan et al. [66] found that the optimum amount of coconut fiber in concrete is 0.25%, which provided approximately 19% improvement in compressive and flexural strength at 28 days. It was verified in this study that the greater the amount of coconut fiber in the concrete, the lower the tensile strength, and that with the presence of fibers in the concrete, there is lower workability. In this study, the compressive, flexural, and tensile strengths of coconut fiber-reinforced concrete were obtained and evaluated, considering variations in the amount of coconut fiber of 0%, 0.25%, 0.5%, 0.75%, and 1.0% by weight of aggregates.

Since the fibers added by Sekar and Kandasamy [67] were added on a volume basis, the aspect ratio (the ratio between the fiber length and its diameter) did not influence the densities of the mixtures. Therefore, the slump values were 8 to 10 cm for conventional concrete and 6 to 10 cm for coconut shell concrete mixtures, in which no difficulties were encountered during the molding of the samples or compaction. With increasing fiber percentage, both workability and density decreased. The authors also concluded that an aspect ratio of 83.33 and a volume fraction of 3% are the best conditions used in conventional mixtures to achieve maximum compressive strength. For mixtures with coconut shells, coconut fiber with an aspect ratio of 66.67 and a volume fraction of 3% are the ideal values for these mixtures to achieve maximum compressive strength.

Regarding the maximum flexural strength, Sekar and Kandasamy [67] found that for conventional concrete mixtures and concrete with coconut shell and coconut fibers, the flexural strengths were 30.63% and 53.66%, respectively, higher than conventional and coconut shell mixtures without coconut fiber. The aspect ratio of coconut fibers significantly improves flexural strength, and the flexural strength increases with increasing aspect ratio.

Another verification done by Nehvi and Kumar [56] was for compressive strength. The study confirmed that coconut fiber increased the compressive strength of concrete when 3% fiber was used. Coconut fiber was used in percentages ranging from 0% to 5% by volume of concrete. The average compressive strength of concrete increased from 27.76 MPa for the control mixture with 0% fiber content to 32.10 MPa for 3% fiber content, showing an increase of 15.6%. The increase in fiber concentration (above 4%) decreased the average compressive strength from 32.1 MPa to 25.36 MPa. The maximum compressive strength was observed with 3% coconut fiber after 28 days (36.4 MPa), followed by 14 days (34.7 MPa), and 7 days (25.2 MPa).

Nehvi and Kumar [56] also analyzed the tensile and flexural strengths. A proportional increase in tensile strength was achieved from 2.65 MPa (conventional concrete mix) to 3.14 MPa with 3% fiber. However, higher concentrations of coconut fiber (above 3%) exhibited adverse effects on tensile strength. The highest concentration (5%) recorded the lowest tensile strength (2.4 MPa), showing a 9.4% decline compared to conventional concrete. The maximum tensile strength (3.56 MPa) was reached after 28 days for the concrete mixture with 3% coconut fiber. As for flexural strength, they recorded an average strength of 6.43 MPa with 3% coconut fiber and 5.7 MPa with 5% coconut fiber. At 28 days of curing the concrete mixture with 3% coconut fiber, the highest value of flexural strength (7.48 MPa) was found. The best concentration (3%) also recorded significant superiority after 7 days and 14 days of curing. The flexural strength increased by 17.9% with 3% coconut reinforcement.

Ahmad et al. [57] analyzed the use of coconut fibers in high-strength concrete considering different lengths of coconut fiber (25, 50, and 75 mm) and contents (0.5%, 1.0%, 1.5%, and 2.0%, by mass) to investigate its mechanical properties considering structural applications. The results of the high-strength concrete reinforced with coconut fiber were compared to the results of the high-strength concrete without coconut fiber but with the same mix design. The authors concluded that the slump and density of the high-strength concrete reinforced with coconut fiber were reduced compared to the high-strength concrete without fiber; by changing the length and percentage of fiber, the slump of the high-strength concrete reinforced with coconut fiber was reduced up to 87.5% and the density was reduced by up to 2.7% compared to high-strength concrete without fiber; the mechanical strengths and water absorption rate of the high-strength coconut fiber-reinforced concrete were improved by using only 1.5% content and 50 mm length.

Regarding durability, Ramli et al. [68] studied the properties of coconut fiber-reinforced concrete in aggressive environments. They reported that incorporating coconut fibers improves durability compared to conventional concrete. The strength and durability of coconut fiber-reinforced concrete under various aggressive environments, such as exposure to seawater and air for various durations, were examined. Durability tests such as carbonation depth, intrinsic permeability, and chloride penetration were performed. The microstructure and mineralogy were also studied by scanning electron microscopy and X-ray diffraction. Due to the addition of coconut fibers, compressive strength and flexural strength were improved by up to 13% and 9%, respectively. In addition to the improved compressive and flexural properties, intrinsic permeability, carbonation depth, and chloride penetration were improved. It was observed from the microstructure and mineralogical studies that in a seawater environment, it affected both the conventional concrete and coconut fiber-reinforced concrete samples. On the other hand, using a smaller amount of coconut fiber may be beneficial in terms of durability. Therefore, it was recommended that a smaller amount of coconut fiber be used to consider durability. By evaluating all tested parameters, the approximate threshold value of coconut fiber is 1.2% (per volume of cement), which would be adequate and beneficial for long-term durability and strength in all tested aggressive environments.

Table 4 summarizes the mechanical performance results of the above investigations.

## 4. Textile Mesh Reinforced Cementitious Composites

Textile-reinforced concrete (TRC) is composed of cement paste, fine aggregates, mineral additions, and reinforcements with long, bidirectional fibers. It is a type of reinforced concrete consisting of a cementitious matrix of low granulometry that involves two- or three-dimensional textile reinforcement made by high-performance fibers, which can be classified as synthetic, metallic, or organic [69]. To produce coir textile mesh, twisted coir yarns are used, and their mechanical performance is highly dependent on the friction between the twisted fibers, determined by the contact area and fiber surface roughness [51].

The mechanical properties of the TRC are affected by the alignment of the textile reinforcement, oriented in the direction of the primary efforts, which guarantees different characteristics to the concretes reinforced with the discontinuous fibers dispersed in the matrix. This technique promotes the orientation of long, high-performance fibers arranged according to the stresses and groups them in the regions most subject to tension. This peculiarity allows better use of the fiber properties and, consequently, enables the replacement of steel bars by a textile reinforcement in tensioned regions [70].

According to Truong and Kim [70], textile-reinforced cementitious composites (TRCCs) are innovative cement-based construction materials using 2D/3D fabrics as continuous reinforcements. Ortolan et al. [69] mention that the type of fabric and its configuration are determined according to the application type and the conditions to which the composite will be subjected. Unidirectional or bidirectional flat fabrics are commonly used in the production of thin laminates as well as structural reinforcement or repair. 3D fabrics are produced to strengthen structural elements such as beams, columns, and plates.

According to De Munck et al. [71], reinforcing a cementitious matrix with a non-corrosive fabric results in a promising material for various applications. This combination of materials has already proven its effectiveness for the recovery and/or structural reinforcement of reinforced concrete and the realization of slender structures such as facade panels and walkways. Carbon fibers, glass, basalt, and even natural fibers reinforce cementitious matrices. During their service lives in outdoor applications, TRCCs are subject to varying climatic conditions: wind, rain, freezing, heat, and more. To ensure the performance of a TRCC throughout its service life, it is essential to understand its long-term behavior under these loading conditions.

According to Truong and Kim [70], there is no standard technical standard or test method for TRCCs, although there are some design guidelines and test method recommendations for TRCCs, including AC 434 and ACI 549.4 R-13. In addition, research on the behavior and application of TRCCs is still limited, although TRCCs have been successfully applied for strengthening lightweight structures and have been gradually replaced by conventional reinforced concrete structures. A greater understanding of the mechanical properties of TRCCs is needed for their practical application. TRCC still presents some deficiencies and uncertainties regarding its properties, particularly textile crosslinking.

Due to the continuous fibers, these composite materials exhibit strain-hardening behavior and a high load capacity, leading to high mechanical performance. In addition, the formation of fine cracks in these composites makes the internal structure less permeable and offers better performance and high durability. Thus, eventually, textile-reinforced composite materials will be less subject to corrosion problems than conventional building materials [72]. 

According to Daskiran et al. [72], textile-reinforced cementitious composites are used as prefabricates that serve as architectural (non-structural) and structural (load-bearing) elements. Exterior cladding panels, parapet walls, and acoustic barriers are the most common architectural applications of TRCCs. In contrast, permanent formwork elements, pedestrian walkway segments, and load-bearing sandwich panels are the most common structural applications of TRCC. High-quality surface finishes, improved mechanical properties, reduced product thickness (in the range of 10–30 mm), and high durability are the advantages of TRCC for the construction industry. Therefore, structural, functional (thermal performance, moisture and sound protection, fire resistance), aesthetic, and long-term (durability) performance are essential considerations in the design of TRCC panel elements. 

Fiberglass or carbon fiber are the most common 2D textiles used to make TRC panels. Basalt, polyphenylene benzobisoxazole (PBO), PVA, and sisal fiber mesh textile materials have also been widely used in recent years. The matrix should have a compressive strength of up to 90 N/mm^2^ and a modulus of rupture of 10 N/mm^2^ to keep the composite crack-free under service conditions [72].

According to De Munck et al. [71], TRCC is characterized by a linear behavior in compression, defined by the characteristics of the mortar, and a nonlinear behavior in tension. This nonlinear tensile behavior has been studied and is characterized by three different stages and some distinct parameters.

Stage I characterizes the uncracked element, which corresponds to the modulus of elasticity of the cementitious matrix, that is, a linear stage in which the stiffness during this stage is strongly dependent on the stiffness of the matrix. In this stage, only the matrix is solicited, whereas, from the first crack, the fibers become effectively solicited, and a significant decrease in stiffness is observed [69].

Stage II begins with the onset of the first crack and its propagation across the width of the specimen, which occurs perpendicular to the minor side of the element. Upon entering stage II, the composite’s stiffness gradually decreases due to the formation of regularly spaced cracks (multiple cracking stages). The condition necessary to reach these multiple cracking stages is related to the fiber volume fraction. A certain amount of fiber must be inserted to achieve strain-hardening and ductile-tensile behavior. The minimum amount of reinforcement is defined by the critical fiber volume fraction, which is dependent on the modulus of elasticity (E) and the ultimate strength of the matrix and the fibers [69,71].

According to Ortolan et al. [69], the spacing of the cracks is determined by the reinforcement and its bonding characteristics with the concrete. Even with the propagation of cracks along the specimen, the load capacity of the cracked composite keeps increasing, resulting in the beginning of stage III. At this stage, there are no more occurrences of cracks; only reinforcement is requested.

This behavior is attributed to the strength and modulus of elasticity of the textile reinforcement, and the stiffness depends exclusively on the stiffness of the textile reinforcement and the fiber volume fraction. Thus, failure occurs when the tensile stress of the reinforcement is reached, characterized as the maximum (peak) strength of the cementitious composite reinforced with textile [69,71].

When the TRCC is asked to bend, a scenario that characterizes the behavior in use because it approximates the real-world conditions of the textile concrete, the matrix compression reactions and reinforcement traction work together to meet the bending requests. Ortolan et al. [69] argue that since the compressive strength of the matrix is higher than the tensile, the most significant demand of TRCC is guided by the tensile zone.

During the telescoping rupture process, only a portion of the yarn’s filaments are active. According to the level of adhesion of the outer filaments to the inner layers, there is a greater or lesser number of simultaneously active filaments. The proportion of active filaments at different loading levels directly influences the rupture mode, which can be fragile or present pseudoductility due to the telescopic rupture process [73].

Nonetheless, the wire can withstand higher loads when there are a greater number of active filaments, according to the authors. However, when these filaments rupture, there is a sudden drop in the supported load, indicating fragile behavior. When the number of active filaments is reduced, the wire exhibits the rupture of the first filaments at a lower load. However, this loss of support capacity occurs more slowly due to the telescopic rupture, indicating a more ductile behavior.

## 5. Coconut Fiber Treatments

According to Gholampour and Ozbakkaloglu [24], due to the high sensitivity of natural fibers to moisture, moisture absorption results in delamination between the matrix and the fiber, reducing the mechanical properties of the composite. This is attributed to the fact that, due to the presence of non-cellulosic components (i.e., pectin, lignin, and hemicelluloses), natural fibers in nature are polar and hydrophilic and therefore create active conditions (i.e., accessibility to hydroxyl (OH) and carboxylic acid groups) for water absorption. According to Tian et al. [25], in an alkaline cementitious medium, the lignin of natural fibers is decomposed, causing a significant degradation in the strength of natural fiber-reinforced concrete. Natural fibers may retain only about 20% of their original tensile strength after being attacked by NaOH or Ca(OH)_2_ solutions.

Tian et al. [25] mention that several studies have been conducted to improve the mechanical properties of natural fiber and, consequently, the physical and mechanical properties of natural fiber-reinforced cementitious composites. The different fiber modification methods and their impacts on the properties of natural fibers or natural fiber-reinforced composites are presented below.

Tian et al. [25] indicated two proposals to reduce alkali attacks on fibers. One involved using pozzolanic materials, such as rice husk ash, flax particles, fly ash, fine powdered brick waste, slag, and sugarcane bagasse ash, to replace part of the cement and thus reduce alkali attack on natural fibers. Using pozzolanic materials to replace some of the cement can dilute the matrix’s alkali concentration, reducing alkali attack on natural fibers and preserving the composite’s mechanical properties. The other approach involved treating the natural fibers chemically or thermally. Chemical or thermal treatments of natural fibers can improve their physical and mechanical properties. As a result, treated natural fibers can show better durability even after long-term alkali attacks, with great potential to improve the long-term mechanical properties of cementitious composites.

In addition, Gholampour and Ozbakkaloglu [24] cite that differences in environmental conditions, such as the amount of sun, rain, soil conditions, and the amount of water the plant receives during the growth period, as well as processing and production conditions, can also affect the performance of natural fibers. Therefore, the performance and properties of natural fibers can be different at each harvest time and even within the same crop population. The other limiting factor for using natural fibers in composites is their low thermal stability. However, physical and chemical modifications can solve the problems concerning using natural fibers in the composite.

The physical treatments on fibers modify their structural and surface properties without transforming their chemical composition by increasing the mechanical adhesion between the natural fiber and the matrix, improving the interface without changing the chemical properties of the fibers [24]. Among the physical treatments are hornification, corona, cold plasma, ultraviolet rays, and heat treatments with electron radiation. Hornification is the application of wetting and drying cycles to fibers, which results in changes in water retention as well as mechanical behavior. After the wetting and drying cycles, the fiber cell walls collapse, resulting in modifications of their structure, such as a reduction in the lumen diameter and deformations in the fibrocellular walls [74].

According to Gholampour and Ozbakkaloglu [24], the surface energy of cellulose fibers with corona treatment is altered to improve the compatibility between the hydrophilic fiber and the matrix. A high voltage at a low temperature is used to generate an atmospherically pressured plasma. The surface energy of the cellulose fibers is changed in the plasma treatment method by a surface modification technique similar to that of the corona treatment. However, in plasma treatment, the type, flow, pressure, and concentration of the gas are controlled; in corona treatment, they are not. The UV treatment method increases the polarity of the fiber surface, leading to improved fiber wettability and increased strength in the composite.

Chemical modification of natural fibers improves the adhesion between the matrix and the natural fibers through chemical reactions. According to Gholampour and Ozbakkaloglu [24], several studies have been conducted to understand the effect of chemical treatment on natural fibers. The hydrophilic nature of natural fibers and the hydrophobic nature of matrices are considered two different phases, resulting in weak bonds at the interfaces of natural fiber composites. Chemical treatment of natural fibers decreases the fibers’ inherent hydrophilic behavior and improves the matrix and fiber adhesion properties. Chemical treatment methods include alkaline or mercerization by silane, acetylation, benzoylation, peroxide, maleate coupling agents, sodium chlorite, and fungal treatments, among others [74,75]. 

The most used method is the alkaline treatment, or mercerization, for its low cost [24,74,75]. This treatment removes much hemicellulose, lignin, and soluble materials from the fiber, roughening its surface. The alkali treatment approach is one of the simplest, most economical, and most effective methods to improve the adhesion properties of natural fibers to the matrix. In this method, the cellulosic molecular structure of natural fibers is modified using sodium hydroxide (NaOH). The alkaline treatment increases the speed of fiber fragmentation and disaggregation. The order orientation of the highly compacted crystalline cellulose is altered by creating amorphous regions in which the cellulose micromolecules are separated and the spaces are filled with water molecules. The alkali sensitive OH^−^ groups are broken down and moved out of the fiber structure. Therefore, the number of hydrophilic OH^−^ groups decreases, the fiber’s resistance to moisture increases, and a certain amount of hemicelluloses, lignin, pectin, wax, and oil are removed. Fiber surfaces become clean and uniform, which improves the ability to transfer stress between cells. An optimum alkali concentration should be obtained to avoid extra delignification of the fibers, but higher concentrations can weaken and damage the fibers. With an optimum alkali concentration, the fiber diameter is decreased, resulting in better bonding due to increased effective fiber surface area and aspect ratio (length/diameter). Alkali treatment is the most efficient approach for exposing natural fibers to cellulose and can maintain the native hydrophilic characteristic of green coconut fibers and increase their thermal stability. 

According to Tian et al. [25], this treatment can remove the amorphous part of the fibers, waxes, hemicelluloses, and pectins. After the treatment, natural fibers become rougher and adhere better to the matrix. In addition, the composites reinforced with NaOH-treated fiber also showed a very ductile behavior with better fiber-matrix adhesion.

In order to protect and improve fiber-matrix adhesion, several authors have been performing surface treatments on the fibers before producing natural fiber-reinforced composites.

Tian et al. [25] report that it should be noted that although pre-modification is generally required for natural fibers in order to improve their mechanical properties since natural fibers are mainly agricultural waste, the cost of natural fiber, including the cost of modification, is believed to be much lower than the cost of other fibers, such as steel, glass, or synthetic fibers. Because of the variation in natural fiber types and the modification methods used for different types, it is not easy to assess the total cost of modified natural fibers. Furthermore, in the laboratory, fiber modification is usually done on a small scale, so the cost is much higher than in mass industrial production. Therefore, an evaluation of the cost of natural fibers modified in the laboratory will not apply to industrial production.

According to Martins and Sanches [76], the standard procedures applied to coconut fibers are cleaning and softening to remove lignin and thus improve tactility, and spinning partially. For commercial reasons, the bleaching procedure is advisable after the alkali treatment. Although sodium hydroxide (NaOH) is the most common softening agent, it causes fiber strength loss. A typical coconut bleaching recipe consists of a 1:20 ratio of the solution containing 8 g/L hydrogen peroxide (H_2_O_2_) and 5 g/L sodium silicate. The liquid is kept under constant stirring at a temperature of 80–90 °C for 60 min, rinsed in cold water, and dried in the shade. The spinning of coconut fiber is complicated to perform industrially. This procedure is often performed manually due to the fibers’ inability to generate continuous roving. Washing is a preparatory process in cellulosic fiber textiles to remove impurities such as organic debris, natural wax, and pectin. It prepares the textile surface for satisfactory performance in the subsequent steps, such as dyeing and finishing.

Andiç-Çakir et al. [77] reported that mortar with treated fiber had slightly higher compressive and flexural strengths after immersing coconut fiber in a 5% NaOH solution for 2 h, compared to untreated coconut fiber.

Kochova et al. [78] submitted coconut fiber to be used in cementitious composites to a pre-treatment leaving the fiber for 24 hours in a Ca(OH)_2_ solution at 20 °C. And then we analyzed two situations: the first was already taking the pre-treated fiber to the oven at 60 °C, and the second was taking the pre-treated fiber to the tap once and then taking it to the oven at 60 °C. FT-IR spectroscopy (Fourier transform infrared spectroscopy) was used as a semi-quantitative analysis. The main problem known from the literature is hemicellulose, which affects cement hydration. In addition, wax and other impurities on the surface can also affect the cement-fiber interaction.

As for the mineralogical measurements of coconut fibers, the amorphous content of the fibers decreases after treatment, mainly due to the removal of hemicellulose and other amorphous constituents, while cellulose remains practically unchanged. No Ca(OH)_2_ or CaCO_3_ remaining from the pre-treatment can be detected in the fibers. When an alkaline treatment is applied, it impacts the cellulose’s morphology and molecular and supramolecular properties. Consequently, the fibers are stiffer, more accessible, and exhibit changes in crystallinity and pore structure [78].

Also, according to Kochova et al. [78], a characterization of the fiber surface is performed to evaluate the effect of pretreatment on coconut fiber morphology. Pretreatment led to morphological changes due to the removal of compounds such as wax and hemicellulose. It was also noticeable that prolonged pretreatment (24 h) with washing can be detrimental to some fibers.

The treatment of natural fibers, especially coconut fiber, still generates many uncertainties because besides the variation of impurities in the fiber, which affect fiber/matrix adhesion, there are still different results for the various types of treatments, and there is variation in the percentages of fiber analyzed. Given these uncertainties, this corroborates the statement that there is still no definition of the subject and no standardization.

## 6. Conclusions

Adding plant fibers, especially coconut fibers, can reduce the environmental impact of cement-based building materials. Coconut fibers are inexpensive, readily available, recyclable, and of low density. Under stress, they exhibit stress-strain behavior. Previous investigations have shown that with increased elongation of coconut fiber, better physical and mechanical properties can be imparted to cementitious composites.

However, a better understanding of the physical and chemical characteristics of the fiber is needed to make its use feasible. This exploratory research sought to establish a comparison between the physical and mechanical performance of cementitious matrices reinforced with coconut fibers and coconut fiber textile meshes, having as a reference several studies available in the literature.

## 7. Future Perspectives

Throughout the paper, it was realized that more studies are needed to elucidate the potential effect of coconut fiber in composites under different parameters such as matrix water/cement ratio, geometry, alignment, ideal contents, pretreatment of matrix and fiber, combined use of fiber with supplementary cementitious materials, rheological properties, drying shrinkage, long-term durability after natural exposure and aggressive environments, data from non-destructive testing, behavior at high temperatures, application in precast elements, application in paving works, behavior via the finite element method when subjected to different stresses, and evaluate the life cycle of these cement-based materials.

## Figures and Tables

**Table 1:** Physical and mechanical properties of natural fibers.

Fiber Type	Density (g/cm^3^)	Length (mm)	Diameter (µm)	Tensile Strength (MPa)	Tensile Modulus (GPa)	Elongation (%)	Micro-Fibrillar Angle (Deg)	Moisture Content (wt%)	Ref.
Bagasse	1.25	10–300	10–34	222–290	17–27.1	1.1	-	-	[28,29]
Bamboo	0.6–1.1	1.5–4	25–40	140–800	11–32	2.5–3.7	-	-	[30]
Banana	1.35	300–900	12–30	500	12	1.5–9	-	8.7–12	[31,32]
Coir	1.15–1.46	20–150	10–460	95–230	2.8–6	15–51.4	30–49	8.0	[31,32,33]
Cotton	1.5–1.6	10–60	10–45	287–800	5.5–12.6	3–10	-	7.85–8.5	[32,34]
Curaua	1.4	35	7–10	87–1150	11.8–96	1.3–4.9	-	-	[35]
Flax	1.4–1.5	5–900	12–600	343–2000	27.6–103	1.2–3.3	5–10	8–12	[32,35]
Hemp	1.4–1.5	5–5.5	25–500	270–900	23.5–90	1–3.5	2–6.2	6.2–12	[32,35]
Henequen	1.2	-	-	430–570	10.1–16.3	3.7–5.9	-	-	[32]
Jute	1.3–1.49	1.5–120	20–200	320–800	8–78	1–1.8	8.0	12.5–13.7	[32,35,36,37,38,39]

**Table 2:** Chemical composition of natural fibers.

Fiber Type	Cellulose (wt%)	Lignin (wt%)	Hemicellulose (wt%)	Ref.
Bagasse	32–55.2	19–25.3	16.8	[28,29]
Bamboo	26–65.0	5–31.0	30	[30]
Banana	63–67.6	5	10–19.0	[31,32]
Coir	32–43.8	40–45.0	0.15–20.0	[31,32,33]
Cotton	82.7–90	<2.0	5.7	[32,34]
Curaua	70.7–73.6	7.5–11.1	9.9	[35]
Flax	62–72	2–5.0	18.6–20.6	[32,35]
Hemp	68–74.4	3.7–10.0	15–22.4	[32,35]
Henequen	60–77.6	8–13.1	4–28.0	[32]
Jute	59–71.5	11.8–13.0	13.6–20.4	[32,35,36,37,38,39]

**Table 3:** Moisture balance of natural fibers.

Fiber Type	Balance Humidity (%)
Coir	10
Flax	7
Jute	12
Bamboo	8.9
Hemp	9
Bagasse	8.8

**Table 4:** Synthesis of the mechanical results of the investigated studies.

Optimum Replacement Content (%)	Compression Strength (MPa)	Tensile Strength (MPa)	Flexure Strength (MPa)	Ref.
3	36.4	3.56	8.25	[56]
1.5	61.34	4.90	12.40	[57]
1.5	34.03	4.06	8.50	[58]
1.5	51.00	-	-	[59]
1.25	32.90	~2.25	~8.00	[60]
1.00	~50.00	-	5.50	[63]
0.25	~27.50	~2.50	~6.00	[66]
3	43.80	4.30	5.10	[67]

## Data Availability

Not applicable.
